# Accuracy of parent- and self-reported height and weight data in children: a systematic review

**DOI:** 10.1093/eurpub/ckag175

**Published:** 2026-09-28

**Authors:** Jacqueline A Deurloo, Paula van Dommelen, Renate van Zoonen

**Affiliations:** TNO, Child Health, Leiden, The Netherlands; GGD Hollands Noorden, Youth Health Care, Alkmaar, The Netherlands; TNO, Child Health, Leiden, The Netherlands; TNO, Child Health, Leiden, The Netherlands

## Abstract

Growth monitoring is a key component of preventive youth healthcare (YHC) in the Netherlands and many other countries. Parent- and self-reported height and weight measurements can be used to enhance flexibility and parental involvement, especially during remote visits. While these data are practical, concerns exist about their accuracy, particularly for individual assessment. This literature review aimed to determine the accuracy of parent- or self-reported height and weight data for use in YHC. A systematic review was conducted, including studies from the past 13.5 years comparing parent- or self-reported and measured height and weight data in children. Data on correlation coefficients and mean differences were extracted. Meta-analyses were performed by reporting source, age group, and sex. Twenty-nine studies were included. Correlations between reported and measured values ranged from poor to almost perfect. Parent-reported data were more accurate for children aged 4–11 years than for those aged 0–3 years; self-reported data were more accurate in adolescents aged 12–18 years than for those aged 4–11 years. Systematic underestimation of height and weight was observed in parent-reported data for those aged 4–11 years. Self-reported data showed similar trends, with accuracy increasing with age. We conclude that parent- and self-reported height and weight data lack sufficient accuracy for individual growth monitoring in YHC. If used, the data source should be clearly indicated in health records. Further research is needed to improve the accuracy of reported measurements for potential use in Dutch YHC.

## Introduction

Growth monitoring is a well-known part of preventive youth healthcare (YHC) in the Netherlands and many other countries. Regular assessment of height and weight data is important for identifying growth-related disorders, but also for detecting underweight, overweight, and obesity as a risk factor [1–4].

To increase flexibility in scheduling and promote parental involvement, YHC has been exploring the use of parent- or self-reported height and weight measurements. These self-reported data may complement professionally obtained data, or serve as substitutes during remote visits, such as telephone or digital appointments [2, 5, 6].

However, there is debate about the usability and accuracy of these measurements. The self-reported data are cheap, easy to use in large-scale population screenings and provide practical data in situations where direct measurement is not feasible [3, 7, 8]. Also, correlations with measured data are often high, especially for weight [3]. However, parents and young people tend to underestimate weight and slightly overestimate height, which is why body mass index (BMI) and the prevalence of overweight are often underestimated [3, 7, 9–11]. This can result in significant misclassification at the individual level.

While a previous systematic review has shown that self-reported height and weight data are often strongly correlated with measured values, concerns remain about systematic reporting bias and individual-level accuracy [3]. Furthermore, parent-reported measurements, age-specific differences, and the growing evidence on remote growth monitoring have received limited attention. This review addresses these gaps by separately analyzing parent- and self-reported data across age groups and sex and by evaluating their suitability for individual growth monitoring in YHC.

This literature review aimed to determine whether parent- or self-reported height and weight data in children were sufficiently accurate for individual growth monitoring within YHC. In addition, we investigated at what age parent- or self-reported data may be considered appropriate for use.

## Methods

A systematic literature search from the PubMed and Cochrane databases was conducted in June 2026. We used the patient, intervention, control, outcome framework to guide the search strategy (Table S1).

Prior to the search, a review protocol was developed. The protocol had not been registered beforehand. Studies were eligible if they were (i) written in English or Dutch; (ii) published between Q1 2013 and Q2 2026; (iii) included both parent- or self-reported and measured height and weight data; (iv) reported differences and/or correlation between reported and measured height and weight data; and (v) participants were between 0 and 18 years old. Studies were included regardless of study design and health conditions of participants. Reference lists of all included studies were manually screened to identify additional relevant publications.

The first author (J.D.) independently screened all identified titles and abstracts based on predefined inclusion criteria. Subsequently, the full texts of potentially eligible articles were retrieved and assessed. Any discrepancies or uncertainties regarding the eligibility of a specific study were resolved through consensus-based discussion between two authors (J.D. and R.vZ.). Figure S1 shows the selection process in a Prisma flowchart.

The following data were collected from each study: study design, country, sample size, sex, age, method of obtaining reported data, time interval between reported and measured data, measurement method, correlation coefficients, and the mean and standard deviation (SD) of the differences between reported and measured data for weight, height, and BMI. Correlation coefficients were used to assess the strength and direction of the relationship between reported and measured data. The difference provided an indication of measurement accuracy.

To enable comparison, all reported values were converted to the metric system: inches to centimeters (× 2.54), pounds to kilograms (× 0.4536), and ounces to kilograms (× 0.02834).

Most studies reported the Pearson correlation coefficients to describe the relationship between reported and measured data. Some studies used the intraclass correlation coefficient (ICC) or Lin concordance correlation coefficient (CCC). To enable comparison, all correlation coefficients were categorized into levels ranging from poor to perfect agreement [12–14].

A meta-analysis was performed on the mean differences between reported and measured values for weight, height and BMI, provided that data were available from at least two studies. Analyses were stratified by parent-reported vs. self-reported, by age group (0–3, 4–11, 12–18 y), and by sex. In some studies, results were reported per subgroup but not for the total group. In these cases, the results of the subgroups were used separately in the analyses. Statistical analyses were performed using the metaphor package in R (version 4.4.0, R Foundation for Statistical Computing, Vienna, Austria).

## Results

The search yielded a total of 370 records (see Fig. S1). After screening titles and abstracts, 53 articles were read in full text. Of these, 29 articles met the inclusion criteria and were included in this review. Most articles (*n* = 12) concerned self-reported measurements by children [3, 7, 10, 11, 15–22], 16 on parent-reported measurements [2, 4, 5, 6, 8, 9, 23–32], and one article described both [33]. The studies originated from North America (*n* = 13), Europe (*n* = 7), Australia/Oceania (*n* = 4), Asia (*n* = 4), and South America (*n* = 1).

Most studies were performed in a general population. Some studies used selected populations, including children attending a pediatric endocrine or growth clinic [2, 5].

The age of the children varied from 1 to 19 years. The studies that used parent-reporting concerned children aged 1–18 years, the studies on self-reporting concerned children aged 6–19 years.

Parent- and self-reported height and weight were most commonly collected through questionnaires or surveys, administered either on paper, online, or by telephone. In most studies, respondents provided estimated current height and weight, although several studies accepted or specifically requested recently measured values. A small number of recent studies obtained parent-measured data during live videoconference sessions [6, 31]. The interval between reported and objectively measured values was generally short, ranging from the same day to several weeks. All study characteristics are reported in Table S2.

### Parent-reported measurements

Parent-reported height showed a slight to almost perfect correlation with measured height (Table S3). This correlation seemed to be stronger in children aged 4–11 years compared to those aged 0–3 years. The mean difference showed a large variation, ranging from an underestimation of −5.3 cm to an overestimation of 2.12 cm (both in children 4–11 years). In children aged 0–3 years, the mean difference ranged from −0.8 to 1.14 cm.

For weight, correlations between reported and measured values were poor to almost perfect. Weight was mostly underreported across all age groups, with mean differences ranging from −2.80 kg to 0.45 kg (both in children 4–11years). In children aged 0–3 years, the mean difference ranged from −0.25 to 0.07 kg.

The correlation between reported and measured BMI also showed a very wide range: from poor to very strong. The mean difference also showed variation, with both an underestimation (−0.6 kg/m^2^) and overestimation (1.5 kg/m^2^) observed within the 4–11 years age group.

A meta-analysis was performed on the mean differences between the reported and measured values for height, weight and BMI in children aged 0–3 and 4–11 years. Two studies assessing parent-reported height and weight in children aged 0–3 years were identified, whereas only one study assessed parent-reported BMI. For children aged 4–11 years, 12 studies assessing parent-reported height and weight were identified, and nine assessed parent-reported BMI.

Findings for 0–3 year-olds were inconsistent, with one study reporting a non-significant underestimation and the other a significant overestimation of both height and weight. The pooled estimate showed a slight overestimation of height, with a mean difference of 0.26 (−1.63, 2.15) cm (Fig. 1), and virtually no difference for weight, with a mean difference of −0.04 (−0.30, 0.22) kg (Fig. 1).

**Figure 1:**
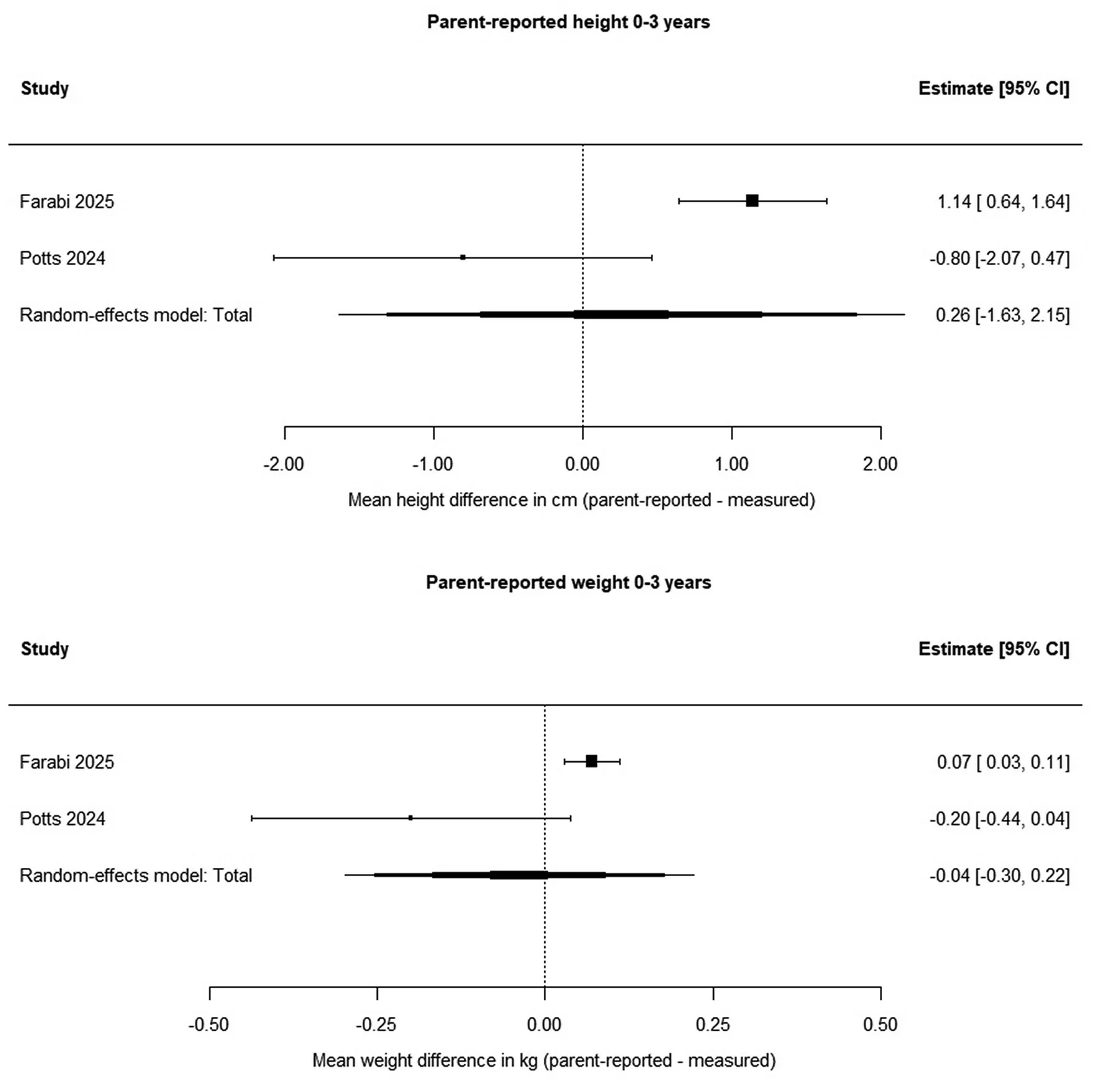
Result meta-analysis of the mean difference between parent-reported and measured height and weight for children aged 0–3 years.

The results revealed a systematic underestimation of height in children aged 4–11-years, with a pooled mean difference of −1.45 (−2.42, −0.48) cm (Fig. 2). Weight was also systematically underestimated, with a pooled mean difference of −0.41 (−0.91, 0.10) kg (Fig. 2). The pooled mean difference in BMI was 0.37 (0.13, 0.61) kg/m^2^ (Fig. 2).

**Figure 2:**
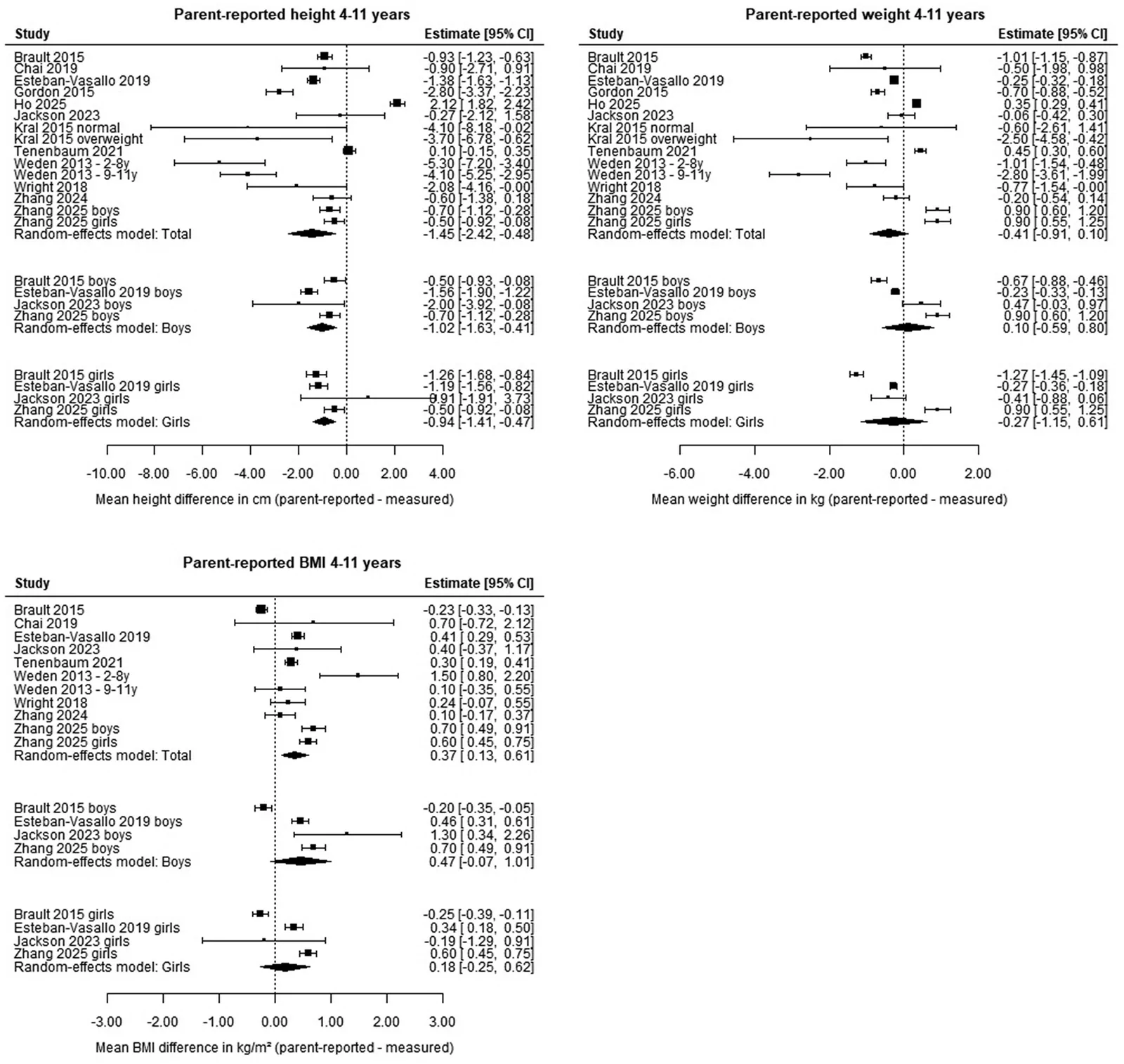
Result meta-analysis of the mean difference between parent-reported and measured height, weight, and BMI for children aged 4–11 years.

Four studies reported sex-specific results for height and weight. For height, the difference in pooled mean difference was negligible [boys −1.02 (−1.63, −0.41) vs. girls −0.94 (−1.41, −0.47)]. For weight, there was a small difference in pooled mean difference, with boys showing a small overestimation [0.10 (−0.59, 0.80)] and girls showing a small underestimation [−0.27 (−1.15, 0.61)]. The pooled mean difference in BMI was slightly higher in boys [0.47 (−0.07, 1.01)] compared to girls [0.18 (−0.25, 0.62)].

### Results for self-reported measurements

Among children and adolescents aged 4–18 years, self-reported height showed a poor to almost perfect correlation with measured values (Table S4). These correlations tended to be stronger in adolescents aged 12–18 years compared to children aged 4–11 years, and in studies with larger sample sizes. The mean difference showed a large variation, with values ranging from an underestimation of −3.23 cm (12–18 years) to an overestimation of 1.9 cm (4–11 years).

For weight, the correlations between reported and measured weight were moderate to almost perfect. The mean difference ranged from −3.28 kg to 1.26 kg (both in children 12–18 years).

The correlation between self-reported and measured BMI was poor to almost perfect. The mean difference showed variation, ranging from an underreporting of −0.87 kg/m^2^ to an overreporting of 0.048 kg/m^2^, both within the 12–18 years age group.

A meta-analysis was performed to assess the pooled mean differences between self-reported and measured values for height and weight among 4–11- and 12–18-year-olds, and for BMI among 12–18-year-olds. The analysis was based on two studies for height and weight in 4–11-year-olds, 11 studies for height and weight in 12–18-year-olds, and nine studies for BMI in 12–18-year-olds.

For height in 4-–11-year-olds, the pooled mean difference was −0.74 (−1.56, 0.09) cm (Fig. 3). A systematic small underestimation was visible. Weight was also systematically underestimated, with a pooled mean difference of −1.88 (−3.96, 0.21) kg (Fig. 3).

**Figure 3:**
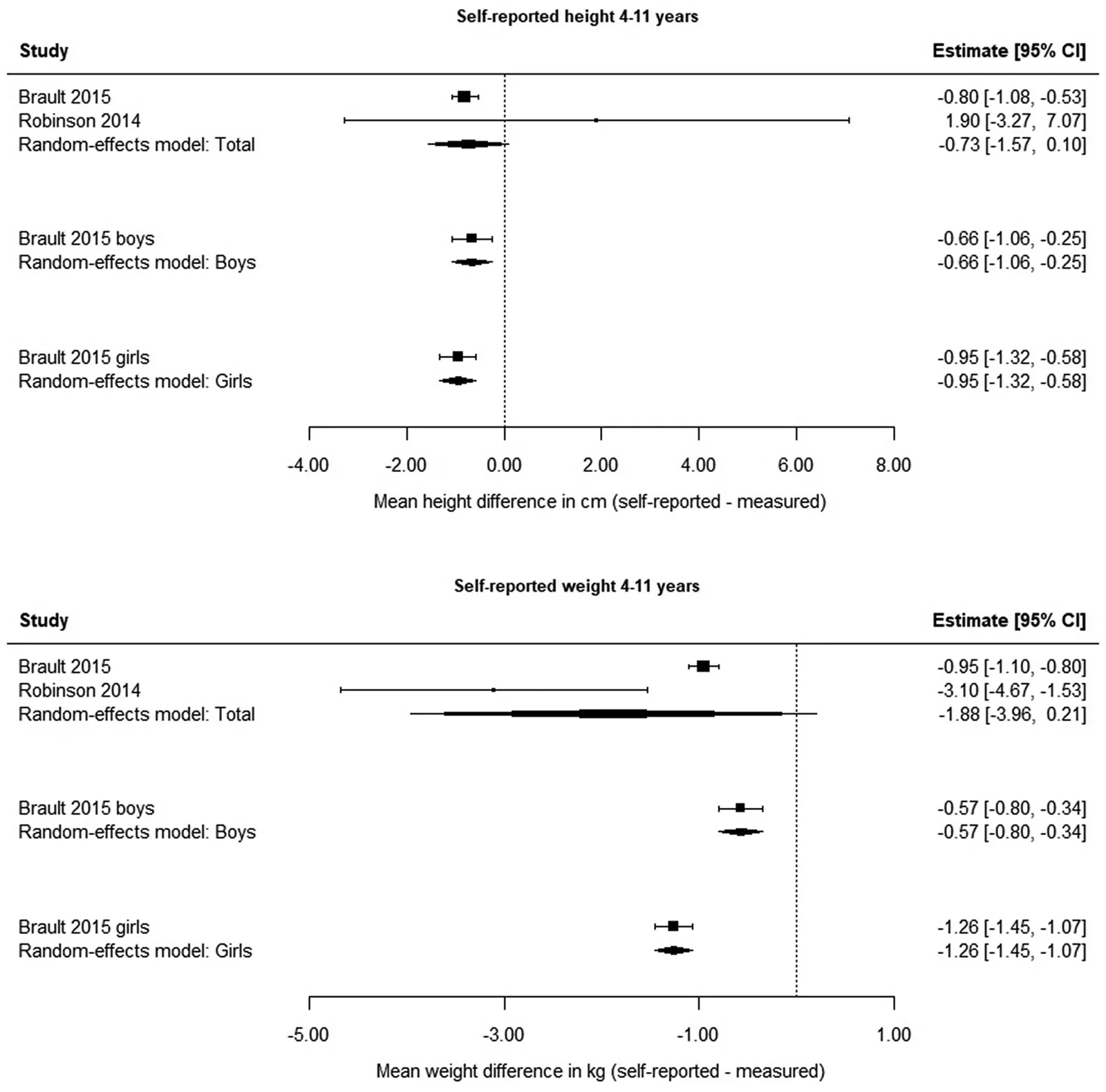
Result meta-analysis of the mean difference between height and weight reported by children (4–11 years) and measured height and weight.

For adolescents aged 12–18 years, the pooled mean difference in height was −0.66 (−1.24, −0.09) cm (Fig. 4). For weight, the pooled mean difference was −0.71 (−1.42, 0.00) kg (Fig. 4). The pooled mean difference in BMI was −0.23 (−0.43, 0.02) kg/m² (Fig. 4).

**Figure 4:**
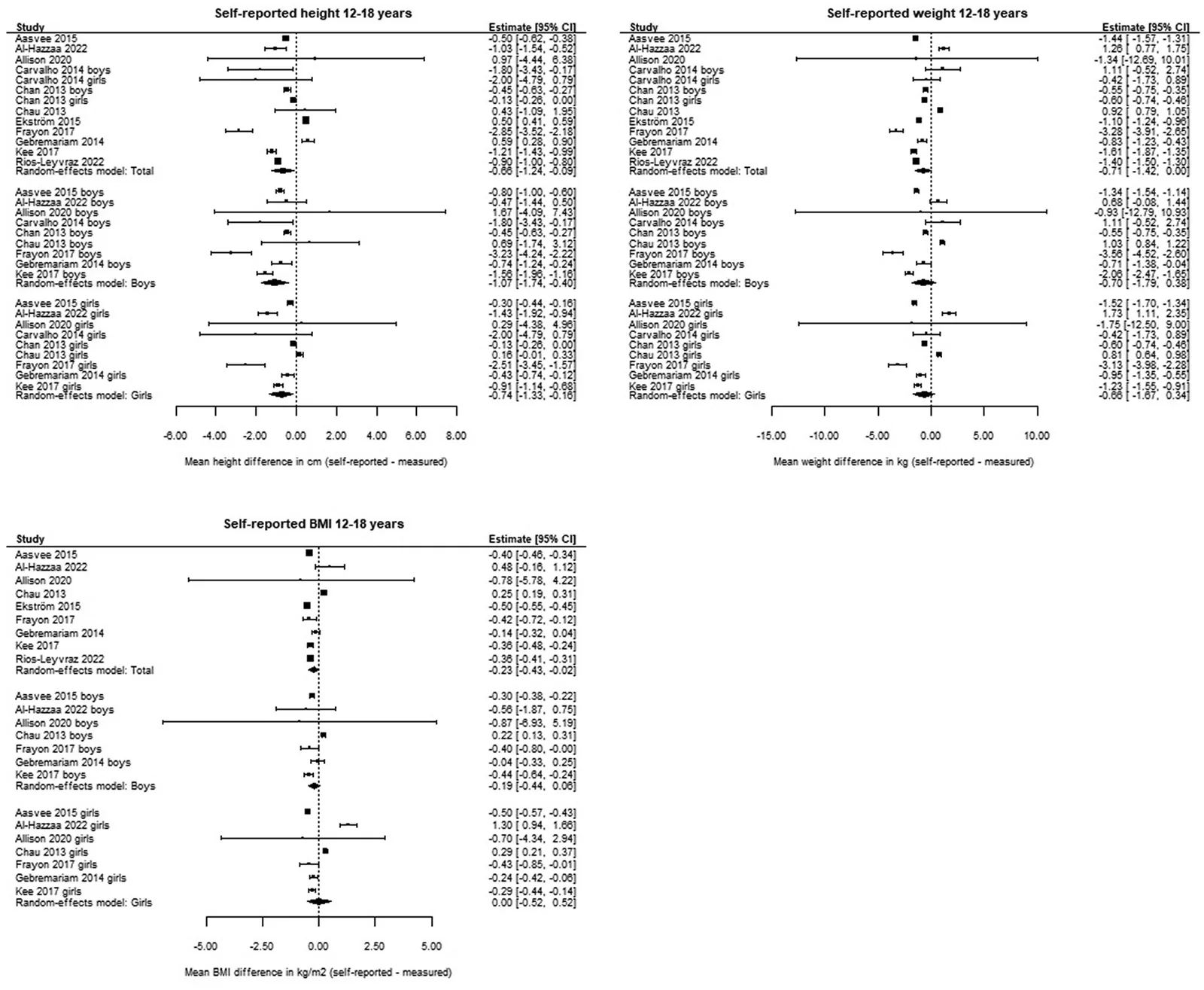
Result meta-analysis of the mean difference between the height, weight, and BMI reported by children (12–18 years) and measured height.

Nine studies reported sex-specific results for height and weight. For height, the pooled mean difference for boys [−1.07 (−1.74, −0.40)] was slightly lower than for girls [−0.74 (−1.33, −0.16)]. For weight, the difference in pooled mean difference was negligible [boys −0.70 (−1.79, 0.38) vs. girls −0.66 (−1.67, 0.34)]. Seven studies reported sex-specific results for BMI. The pooled mean difference for boys [−0.19 (−0.44, 0.06)] was slightly lower than for girls [0.00 (−0.52, 0.52)].

## Discussion

This literature review was conducted to determine the accuracy of values for height and weight reported by parents or children for use in YHC. The findings indicate that the reported weight, height, and BMI showed a very wide range in correlation with measured data, with correlations from poor to almost perfect.

For monitoring individual growth, the mean differences between the reported and measured values and the SD of these differences are also important. The mean differences reflect potential systematic bias, indicating whether individuals tend to consistently over- or underreport their measurements. While the SD of differences provides insight into individual-level accuracy, the mean difference reveals whether there is a structural deviation that could affect interpretation at both the individual and group level. The mean differences show a large variation, with a pooled mean difference reaching of up to −1.45 cm for height (parent-reported, 4–11-year-olds) and −1.88 kg for weight (self-reported, 4–11-year-olds). The study and meta-analysis of Rios-Leyvraz *et al*. [3] also reported high correlations, but also systematic deviations (usually underestimation of weight/BMI). Several studies have shown that even small mean errors can significantly impact BMI classification [10, 16, 27]. Therefore, Kee *et al*. [7] warns that self-reported data should not be used for individual classification. Prior research among adolescents has suggested that the accuracy of self-reported weight may differ by sex [7, 15, 20]. We found only minor sex differences in reporting accuracy, suggesting that age and measurement method may be more important determinants than sex.

Our results suggest that age plays a role in the accuracy of reported data. Self-reported values became more accurate after 12 years, and parent-reported values were more accurate in 4–11-year-olds than in 0–3-year-olds. Our findings are largely in line with earlier studies showing improved accuracy with increasing age, although not all studies observed this pattern [3, 9, 16, 30]. One explanation is that adolescents are more likely to know or recently have measured their own anthropometrics, whereas parents of younger children may rely on memory or visual estimation. On the other hand, adolescents may intentionally or unintentionally misreport their height and weight because of body image concerns, social desirability, or inaccurate perception of body size. Accuracy may therefore depend less on who reports the value and more on whether a recent measurement was available.

Strengths of this study include the inclusion of 29 studies from five continents and the separate analyses by reporting source, age group, and sex. A limitation of this study was the use of different agreement measures across studies, including Pearson correlations, ICCs, and Lin’s CCC, this complicated direct comparisons. We, therefore, categorized the reported coefficients to enable comparison. As CCC is more sensitive to systematic bias, direct comparison between studies is not always straightforward, despite the categorization we used. There was considerable heterogeneity between studies, this may partly be explained by methodological differences. Included studies varied in data collection mode (paper questionnaires, online surveys, telephone interviews, and video consultations), whether respondents estimated current values or reported recent measurements, and the time interval between reported and objectively measured values. Such differences are likely to influence both random error and systematic bias. Also, the assessment of titles, abstracts and full texts was performed by one reviewer, which may have increased the risk of selection bias compared with independent duplicate screening.

Studies have shown that parent-reported values were more accurate when parents measured their child’s weight, and height at home, rather than relying on parental judgements [34]. Further investigation into methods to further improve the accuracy of parent-and self-reported data is warranted. For example, the use of instruction leaflets has shown a small improvement in the correlation between parent-reported and measured weight [35].

Given the growing demand to enhance flexibility and parental involvement within YHC, if YHC-organizations choose to use reported measurements, several precautionary measures may be considered:

- Targeted use in older age groups: the results show that parent-reported data for children aged 4–11 years and self-reported data for adolescents (12–18 years) were more accurate. In these groups, self-measurement/weighing, when properly guided, can be a valuable addition, particularly when in-person consultations are not feasible.
- Use of instructional materials: actively providing clear instruction leaflets or videos to parents and adolescents on how to correctly measure height and weight can improve accuracy [34, 35].
- Combination with professional measurements: self-reported data may be used as a supplement to, or as a bridge between, professional measurements. Recent studies have shown that parent-measured anthropometric data may support clinical assessment when direct measurements are not feasible, although professional measurements remain the preferred reference standard. Therefore, reported measurements may be considered as Supplementary Information between routine professional assessments rather than as a replacement for them. [2, 4, 6]
- Recording the data source: because reported and measured data cannot be considered interchangeable, distinguishing between measured and reported values in health records may facilitate appropriate interpretation of growth trajectories. Possible measurement errors can be taken into account during interpretation, and the risk of misclassification can be reduced.

Additional research into methods to further improve the accuracy of self-measured/weighed data, such as digital tools or feedback systems, is desirable to optimize this approach in the future. In conclusion, although correlations between reported and measured height and weight are often high, systematic bias, and individual-level variability remain substantial. Consequently, parent- and self-reported height and weight data cannot currently replace professional measurements for individual growth monitoring. Further research is needed to explore strategies for improving the accuracy of parent- and self-reported height and weight data, to support the potential use in Dutch YHC.

## Supplementary Material

ckag175_Supplementary_Data

## Data Availability

No new data were generated or analyzed in support of this research. This systematic review shows that parent- and self-reported height and weight data lack sufficient accuracy for individual growth monitoring in youth healthcare. Parent- and self-reported weight, height and BMI data showed a very wide range in correlation with measured data. Parent- and self-reported measurements may be used with caution as supplementary information but should not replace professional measurements for individual growth monitoring.
