# Supplementary material for: Accuracy of parent- and self-reported height and weight data in children: a systematic review
Source: Eur J Public Health. 2026 Sep 28;36(5):ckag175. doi: 10.1093/eurpub/ckag175 (PMC13618880; doi:10.1093/eurpub/ckag175)
Supplement: Supplementary file 1 [file ckag175_supplementary_data.zip › ejph-2025-12-om-1035-File006.docx]

*Table S1: The search strategy used for systematic literature search*.

| 1 | (((((((Anthropometr*[Title/Abstract])) OR (anthropometric*[Title/Abstract])) OR ("body height"[Title/Abstract])) OR ("body weight"[Title/Abstract])) OR "Body Mass Index"[Title/Abstract]) OR (((((Anthropometry[MeSH Terms])) OR (body height[MeSH Terms])) OR (body weight[MeSH Terms])) OR (Body Mass Index[MeSH Terms]))) AND ((self-report[MeSH Terms]) OR ("self-report*"[Title/Abstract])) |
| --- | --- |
| 2 | (((Reproducibility of Results[MeSH Terms]) OR (Data Accuracy[MeSH Terms])) OR (Observer Variation[MeSH Terms])) OR (sensitivity and specificity[MeSH Terms]) |
| 3 | "Acceptability"[Title/Abstract] OR "Feasibility"[Title/Abstract] OR "reliability of results"[Title/Abstract] OR "data accuracy"[Title/Abstract] OR "observer variation"[Title/Abstract] OR "Accuracy"[Title/Abstract] OR "precision"[Title/Abstract] |
| 4 | #2 OR #3 |
| 5 | ((Primary Health Care[MeSH Terms]) OR (public health[MeSH Terms])) OR (community health services[MeSH Terms]) |
| 6 | (((Secondary health care[Title/Abstract]) OR (preventive health care[Title/Abstract])) OR (primary care[Title/Abstract])) OR (health care services[Title/Abstract]) |
| 7 | #5 OR #6 |
| 8 | #1 AND #4 AND #7 |
| 9 | #8 AND ((2013:3000/12/12[pdat]) AND (dutch[Filter] OR english[Filter]) AND (allchild[Filter])) |
