# Supplementary material for: Accuracy of parent- and self-reported height and weight data in children: a systematic review
Source: Eur J Public Health. 2026 Sep 28;36(5):ckag175. doi: 10.1093/eurpub/ckag175 (PMC13618880; doi:10.1093/eurpub/ckag175)
Supplement: Supplementary file 1 [file ckag175_supplementary_data.zip › ejph-2025-12-om-1035-File008.docx]

*Supplementary file 3: study characteristics.*

| ***Study***  ***(first author, year)*** | ***Study design*** | ***Country*** | ***Sample size (n)*** | ***Age***  ***Range or mean (SD)*** | ***Method of obtaining reported data*** | ***Time interval between reported and measured data*** |
| --- | --- | --- | --- | --- | --- | --- |
| ***Parent-reported*** | | | | | | |
| Anthony, 2020 | Cross-sectional study | United States | 234 | 3.4 (0.8) years  2-4 years | Telephone survey  Measured data | < 1 month |
| Brault, 2015 | Longitudinal cohort study | Canada | 807 | 10.3 (1.2) years | Survey  Estimated and/or measured data | Not reported |
| Chai, 2019 | Randomized Controlled Trial | Australia | 42 | 4-11 years  Mean 8.5 years | Online survey  Estimated and/or measured data | Not reported |
| Cheng, 2022 | Randomized Controlled Trial | Australia | 234 | 23.9 (0.4) months | Telephone survey  Estimated and/or measured data | 10.3 (6.9) days |
| Esteban-Vasallo, 2019 | Cross-sectional study | Spain | 2914 | 47-59 months | Reported during visit  Estimated data | Same day |
| Farabi, 2025 | Cross-sectional study | United States | 37 | 21.7 (6.8) months | Measured during live videoconference | Same day |
| Gordon, 2015 | Cross-sectional study | United States | 1053 | 7.5 (3.1) years | Survey  Estimated data | Same day |
| Hazrati, 2016 | Longitudinal cohort study | United States | 185 | 12 months  Mean/SD/range not reported | Survey  Estimated and/or measured data | < 2 months |
| Ho, 2025 | Experimental diagnostic study | United States | 199 | 6.3 (5.4) years | Online survey Measured data | 3.2 (3.1) days |
| Jackson, 2023 | Cross-sectional study | Australia | 89 | 2-6 years (mean 4.7)  SD not reported | Survey (online or telephone)  Estimated and/or measured data | Same day |
| Kral, 2015 | Cross-sectional study | United States | 61 | 5-12 years | Telephone survey  Estimated and/or measured data | Not reported |
| Potts, 2024 | Randomized Controlled Trial | United States | 55 | 46.4 (11.0) months | Online survey Estimated and/or measured data | 16.9 (25.3) days |
| Tenenbaum, 2021 | Retrospective observational study | Israel | 107 | 3-18 years  10.2 (3.7) years | Reported during visit  Measured data | 1 day |
| Weden, 2013 | Cross-sectional study | United States | 8665 | 2-13 years | Survey  Estimated and/or measured data | Not reported |
| Wright, 2018 | Longitudinal cohort study | United States | 616 | 9.0 (1.5) years  6-12 years | Telephone survey  Estimated and/or measured data | 28 (43.9) days |
| Zhang, 2024 | Cross-sectional study | United States | 33 | 6.6-11.9 years  9.3 (1.7) years | Measured during live videoconference | − 2.2 ± 3.3 (SD) days |
| Zhang, 2025 | Cross-sectional study | China | 132 | 6-12 years  Mean/SD not reported | Live interview  Measured data | < 7 days |
| ***Self-reported*** | | | | | | |
| Aasvee, 2015 | Cross-sectional study | Estonia | 3379 | 11-15 years | Survey  Estimated data | Same day |
| Al-Hazzaa, 2022 | Cross-sectional study | Kuwait | 706 | 16.5 (0.9) years | Survey  Estimated data | Same day |
| Allison, 2020 | Cross-sectional study | United States | 412 | 16.3 (7.1) years | Survey  Estimated data | Same day |
| Brault, 2015 | Longitudinal cohort study | Canada | 842 | 10.3 (1.2) years | Survey  Estimated and/or measured data | Not reported |
| Carvalho, 2014 | Cross-sectional study | Brazil | 62 | 12-19 years | Telephone survey  Estimated data | < 1 week |
| Chan, 2013 | Cross-sectional study | Hong Kong | 1614 | 13.3 (3.3) years | Survey  Estimated and/or measured data | 1-2 weeks |
| Chau, 2013 | Cross-sectional study | France | 1559 | 13.0 (1.3) years | Survey  Estimated data | Same day |
| Ekström, 2015 | Longitudinal cohort study | Sweden | 1698 | 16.5 (0.3) years | Survey (online or paper)  Estimated data | 4.6 (1.7) weeks |
| Frayon, 2017 | Cross-sectional study | New Caledonia | 665 | 13.6 (1.5) years | Online survey  Estimated data | ≤ 2 days |
| Gebremariam, 2014 | Intervention study | Norway | 828 | 12.9 (0.3) years | Survey  Estimated and/or measured data | Same day |
| Kee, 2017 | Cross-sectional study | Malaysia | 663 | 13-17 years | Survey  Estimated and/or measured data | Same day |
| Rios-Leyvraz, 2022 | Cross-sectional study | Switzerland | 2616 | 12.2 (0.5) years | Survey  Estimated and/or measured data | Same day |
| Robinson, 2014 | Cross-sectional study | United States | 92 | 10.2 (0.9) years | Survey  Estimated data | 1 day |
