# Supplementary material for: Accuracy of parent- and self-reported height and weight data in children: a systematic review
Source: Eur J Public Health. 2026 Sep 28;36(5):ckag175. doi: 10.1093/eurpub/ckag175 (PMC13618880; doi:10.1093/eurpub/ckag175)
Supplement: Supplementary file 1 [file ckag175_supplementary_data.zip › ejph-2025-12-om-1035-File009.docx]

*Supplementary file 4: results for parent-reported measurements.*

| ***Studies*** | ***Mean age children (years)*** | ***Number of respondents in study*** | ***(Range) mean difference*** | ***Category correlation**** |
| --- | --- | --- | --- | --- |
| *Height* | | | | |
| Farabi 2025, Potts 2024 | 0-3 | <100 | -0.8 - 1.14 cm | Very strong – almost perfect^a,b^ |
| Anthony 2020, Cheng 2022, Hazrati 2016 | 0-3 | 100-<500 | 0.4 cm | Moderate^a,b^ |
|  |  |  |  |  |
| Chai 2019, Jackson 2023, Kral 2015, Zhang 2024 | 4-11 | <100 | -4.1 - -0.27 cm | Fair - almost perfect^a,b,c^ |
| Ho 2025, Tenenbaum 2021, Zhang 2025 | 4-11 | 100-<500 | 0.1 – 2.12 cm | Almost perfect^b^ |
| Brault 2015, Esteban-Vasallo 2019, Gordon 2015, Weden 2013, Wright 2018 | 4-11 | ≥500 | -5.3 - -0.93 cm | Slight-very strong^a,b, c^ |
| *Weight* | | | | |
| Farabi 2025, Potts 2024 | 0-3 | <100 | -0.2 – 0.07 kg | Very strong – almost perfect^a,b^ |
| Anthony 2020, Cheng 2022, Hazrati 2016 | 0-3 | 100-<500 | -0.1 kg | Very strong- almost perfect^a,b^ |
| Esteban-Vasallo 2019 | 0-3 | ≥500 | -0.25 kg | Almost perfect^b^ |
| Chai 2019, Jackson 2023, Kral 2015, Zhang 2024 | 4-11 | <100 | -2.5 - -0.06 kg | Substantial - almost perfect^b,c^ |
| Ho 2025, Tenenbaum 2021, Zhang 2025 | 4-11 | 100-<500 | -0.9 - 0.45 kg | Almost perfect^b^ |
| Brault 2015, Gordon 2015, Weden 2013, Wright 2018 | 4-11 | ≥500 | -2.80 - -0.7 kg | Poor-very strong^a,c^ |
| *BMI* |  |  |  |  |
| Potts 2024 | 0-3 | <100 | 0.1 | Fair - moderate^a,b^ |
| Cheng 2022 | 0-3 | 100-<500 | -0.2 | Fair^b^ |
| Esteban-Vasallo 2019 | 0-3 | ≥500 | 0.41 | Moderate^b^ |
| Chai 2019, Jackson 2023, Zhang 2024 | 4-11 | <100 | 0.1 - 0.7 | Poor - slight^b,c^ |
| Tenenbaum 2021, Zhang 2025 | 4-11 | 100-<500 | -0.6 - 0.3 | Not reported |
| Brault 2015, Weden 2013, Wright 2018 | 4-11 | ≥500 | -0.23 - 1.5 | Poor-very strong^a,c^ |

* Correlation based on (a) Pearson, (b) ICC, (c) Lin
