# Supplementary material for: Accuracy of parent- and self-reported height and weight data in children: a systematic review
Source: Eur J Public Health. 2026 Sep 28;36(5):ckag175. doi: 10.1093/eurpub/ckag175 (PMC13618880; doi:10.1093/eurpub/ckag175)
Supplement: Supplementary file 1 [file ckag175_supplementary_data.zip › ejph-2025-12-om-1035-File010.docx]

*Supplementary file 5: results for self-reported measurements.*

| ***Studies*** | ***Mean age children (years)*** | ***Number of respondents in study*** | ***(Range) mean difference*** | ***Category**** |
| --- | --- | --- | --- | --- |
| *Height* | | | | |
| Robinson 2014 | 4-11 | <100 | 1.9 cm | Fair^a^ |
| Brault 2015 | 4-11 | ≥500 | -0.803 cm | Very strong^a^ |
| Carvalho 2014 | 12-18 | <100 | -2.0 - -1.8 cm | Substantial^b^ |
| Aasvee 2015, Al-Hazzaa 2022, Allison 2020, Chan 2013, Chau 2013, Ekström 2015, Frayon 2017, Gebremariam 2014, Kee 2017, Rios-Leyvraz 2022, Rios-Leyvraz 2022 (meta-analysis) | 12-18 | ≥500 | -3.23 - 1.67 cm | Poor- almost perfect^a,b,c^ |
| *Weight* | | | | |
| Robinson 2014 | 4-11 | <100 | -3.1 kg | Very strong^a^ |
| Brault 2015 | 4-11 | ≥500 | -0.953 kg | Very strong^a^ |
| Carvalho 2014 | 12-18 | <100 | -0.419 - 1.113 kg | Almost perfect^b^ |
| Aasvee 2015, Al-Hazzaa 2022, Allison 2020, Chan 2013, Chau 2013, Ekström 2015, Frayon 2017, Gebremariam 2014, Kee 2017, Rios-Leyvraz 2022, Rios-Leyvraz 2022 (meta-analysis) | 12-18 | ≥500 | -3.28 - 1.73 kg | Moderate - almost perfect^a,b,c^ |
| *BMI* |  |  |  |  |
| Brault 2015 | 4-11 | ≥500 | -0.203 | Very strong^1a^ |
| Carvalho 2014 | 12-18 | <100 |  | Substantial - almost perfect^b^ |
| , Aasvee 2015, Al-Hazzaa 2022, Allison 2020, Chan 2013, Chau 2013, Ekström 2015, Frayon 2017, Gebremariam 2014, Kee 2017, Rios-Leyvraz 2022, Rios-Leyvraz 2022 (meta-analysis) | 12-18 | ≥500 | -0.87 – 0.48 | Poor - almost perfect^a,b,c^ |

* Correlation based on (a) Pearson, (b) ICC, (c) Lin
